# Intratumoral and peritumoral CT radiomics in predicting prognosis in patients with chondrosarcoma: a multicenter study

**DOI:** 10.1186/s13244-023-01582-8

**Published:** 2024-01-17

**Authors:** Qiyuan Li, Ning Wang, Yanmei Wang, Xiaoli Li, Qiushi Su, Jing Zhang, Xia Zhao, Zhengjun Dai, Yao Wang, Li Sun, Xuxiao Xing, Guangjie Yang, Chuanping Gao, Pei Nie

**Affiliations:** 1 Department of Radiology, The Affiliated Hospital of Qingdao University, No. 16, Jiangsu Road, Qingdao, 266003 Shandong China; 2 Department of Radiology, Shandong Provincial Hospital Affiliated to Shandong First Medical University, Jinan, Shandong China; 3 GE Healthcare China, Pudong New Town, Shanghai, China; 4 Department of Radiology, The Affiliated Hospital of Shandong University of Traditional Chinese Medicine, Jinan, Shandong China; 5 Scientific Research Department, Huiying Medical Technology Co., Ltd, Beijing, China; 6 Department of Radiology, The First Hospital of Xingtai, No. 376, Shunde Road, Xingtai, Hebei China; 7 Department of Nuclear Medicine, The Affiliated Hospital of Qingdao University, No. 59, Haier Road, Qingdao, 266061 Shandong China

**Keywords:** Chondrosarcoma, Prognosis, Tomography (X-ray computed), Radiomics

## Abstract

**Objective:**

To evaluate the efficacy of the CT-based intratumoral, peritumoral, and combined radiomics signatures in predicting progression-free survival (PFS) of patients with chondrosarcoma (CS).

**Methods:**

In this study, patients diagnosed with CS between January 2009 and January 2022 were retrospectively screened, and 214 patients with CS from two centers were respectively enrolled into the training cohorts (institution 1, *n* = 113) and test cohorts (institution 2, *n* = 101). The intratumoral and peritumoral radiomics features were extracted from CT images. The intratumoral, peritumoral, and combined radiomics signatures were constructed respectively, and their radiomics scores (Rad-score) were calculated. The performance of intratumoral, peritumoral, and combined radiomics signatures in PFS prediction in patients with CS was evaluated by C-index, time-dependent area under the receiver operating characteristics curve (time-AUC), and time-dependent C-index (time C-index).

**Results:**

Eleven, 7, and 16 features were used to construct the intratumoral, peritumoral, and combined radiomics signatures, respectively. The combined radiomics signature showed the best prediction ability in the training cohort (C-index, 0.835; 95%; confidence interval [CI], 0.764–0.905) and the test cohort (C-index, 0.800; 95% CI, 0.681–0.920). Time-AUC and time C-index showed that the combined signature outperformed the intratumoral and peritumoral radiomics signatures in the prediction of PFS.

**Conclusion:**

The CT-based combined signature incorporating intratumoral and peritumoral radiomics features can predict PFS in patients with CS, which might assist clinicians in selecting individualized surveillance and treatment plans for CS patients.

**Critical relevance statement:**

Develop and validate CT-based intratumoral, peritumoral, and combined radiomics signatures to evaluate the efficacy in predicting prognosis of patients with CS.

**Key points:**

• Reliable prognostic models for preoperative chondrosarcoma are lacking.

• Combined radiomics signature incorporating intratumoral and peritumoral features can predict progression-free survival in patients with chondrosarcoma.

• Combined radiomics signature may facilitate individualized stratification and management of patients with chondrosarcoma.

**Graphical Abstract:**

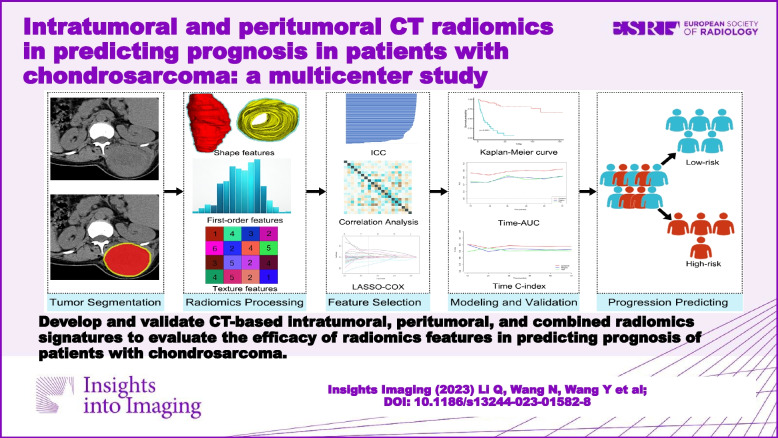

**Supplementary Information:**

The online version contains supplementary material available at 10.1186/s13244-023-01582-8.

## Introduction

Chondrosarcoma (CS) is one of the primary malignant bone tumors characterized by the formation of hyaline cartilage tumor tissue. It accounts for 30% of all primary bone tumors and is the second most common solid bone tumor after osteosarcoma [1]. CS has a low sensitivity to chemotherapy due to its low cell division rate and poor vascular distribution [2, 3]. Currently, surgical excision is the most effective treatment [4]. Although most patients will benefit from surgery, approximately 15–25% of patients will experience local recurrence [5–7]. In the age of precision medicine, accurate prediction of prognosis before surgery is crucial for individualized management of patients and guiding clinical decision-making. However, a reliable survival prediction model for chondrosarcoma is still lacking.

In recent years, several scholars have conducted studies on the prognosis of CS patients. Yan et al. compared the performance of deep learning-based algorithms and conventional methods in predicting the overall survival (OS) of CS patients; nine features including histological type, primary site, tumor size, and other clinical factors were selected for the development of the models. The results showed the best predictive performance of the deep survival model, which integrated the cox proportional hazards model with the neural network, with a C-index of 0.832 for the test database [8]. Song et al. identified six independent prognostic factors based on the Surveillance, Epidemiology, and End Results (SEER) database, including age, histological subtype, grade, surgery, tumor size, and distant metastasis, and incorporated them into the construction of nomograms. They found good agreement between nomogram-predicted survival and actual survival, with C-indexes of 0.803 and 0.829 in the validation cohort in predicting OS- and cancer-specific survival in CS patients, respectively [9]. However, most previous prognostic studies of CS were based on clinical or pathological factors and did not take the addition of imaging features into account.

Medical imaging plays an important role in the management of patients with CS, including the detection, staging, diagnosis, and prediction of outcome [10]. A few scholars have suggested that radiographically observed osteolytic lesions and apparent osteoporosis may be associated with poor prognosis in CS patients, but there have been few relevant studies [11, 12]. Conventional imaging relies on visible features, which provide limited information and might lose a large amount of information associated with tumor heterogeneity [13]. In the period of big data, radiomics, which can noninvasively and quantitatively transform lesion heterogeneity into high-dimensional image features, has been successfully applied to predict the prognosis of tumors originating from the digestive system [14], nervous system [15], and reproductive system [16], thus facilitating precise cancer management and clinical decision-making [17]. To the best of our knowledge, most of the radiomics studies in CS have focused on differential diagnosis and pathological grading [18–21] and were based on intratumoral radiomics features. Peritumoral radiomics features have also demonstrated predictive utility in a variety of cancers [22, 23]. However, the intratumoral and peritumoral radiomics on predicting the outcome of CS has not been evaluated.

The objective of this study was to evaluate the efficacy of the intratumoral, peritumoral, and combined radiomics signatures in predicting the prognosis of patients with CS.

## Materials and methods

### Patients

This retrospective multicenter study was conducted under institutional review board with a waiver of informed consent.

**Fig. 1:**
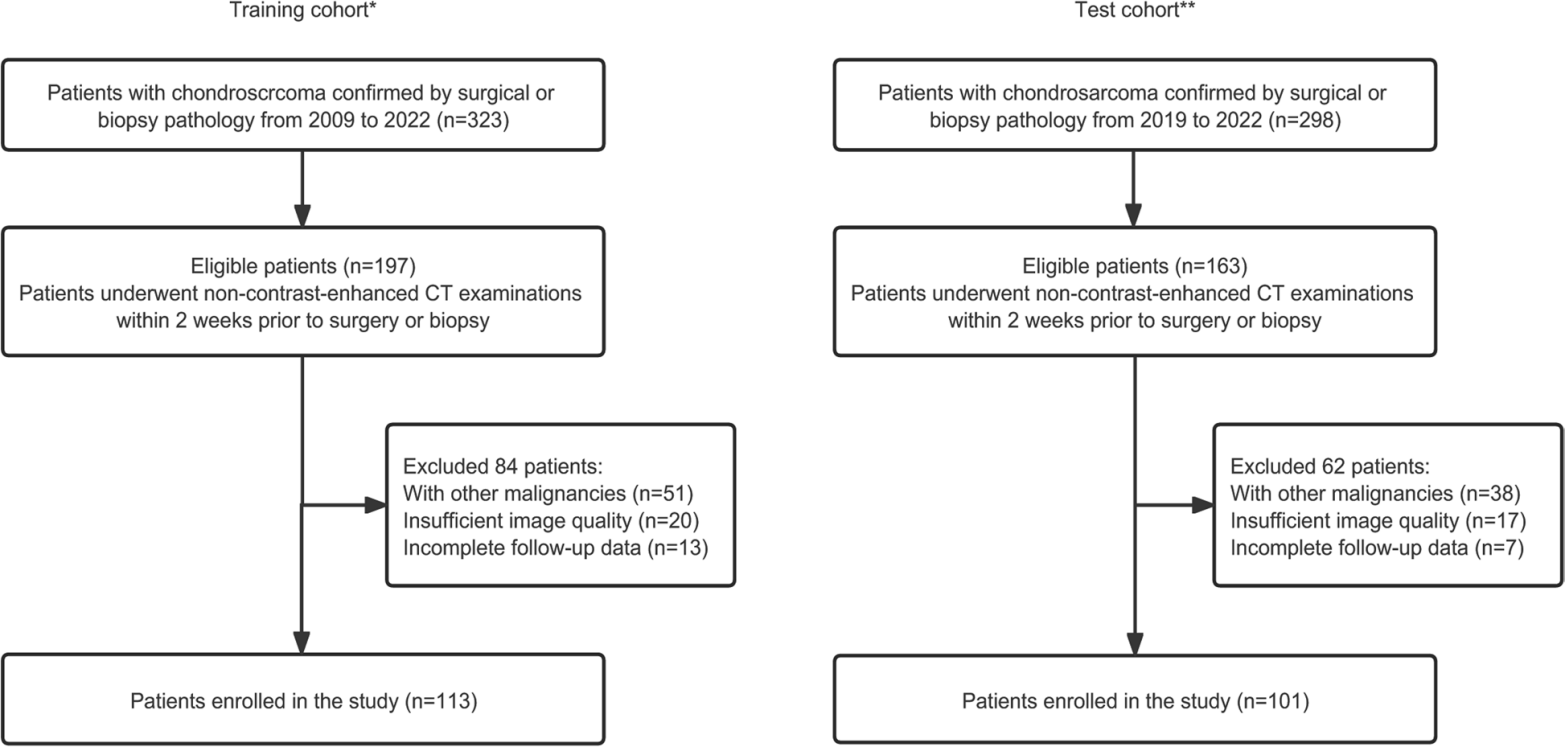
Flow diagram depicting the patient selection process. *The Affiliated Hospital of Qingdao University. **Shandong Provincial Hospital Affiliated to Shandong First Medical University

We searched the institutional pathological databases from January 2009 to January 2022 to select patients diagnosed with CS from surgically resected or biopsy specimens. The inclusion criteria were as follows: (1) CS patients confirmed by surgical or biopsy pathology and (2) CS patients who underwent non-contrast-enhanced CT examinations within 2 weeks prior to obtaining surgical or biopsy pathology. Exclusion criteria included patients with other malignancies, insufficient image quality (images with metallic or motion artifacts), and incomplete follow-up data. Finally, a total of 214 patients from the Affiliated Hospital of Qingdao University (training cohort, *n* = 113) and Shandong Provincial Hospital Affiliated to Shandong First Medical University (test cohort, *n* = 101) were enrolled in this study (Fig. 1).

Clinical and pathological data including age, sex, height, weight, tumor size, tumor site, pathologic grade, and treatment strategy were collected from the electronic medical records.

### Follow-up

Patients were followed up at least every 6–12 months for the first 2 years and then annually. The last follow-up date was December 28, 2022. The follow-up data was obtained through medical records, imaging findings (X-ray, CT, MRI), or telephone. The endpoint of this study was progression-free survival (PFS), defined as the time from the date of diagnosis until local recurrence, distant metastasis, death from any cause, or last follow-up.

### CT data acquisition

Non-contrast-enhanced CT was performed in all patients. Six CT scanners at two centers were used for axial scanning. Detailed CT scanning protocols are shown in Table S1.

### Image segmentation and radiomics features extraction

**Fig. 2:**
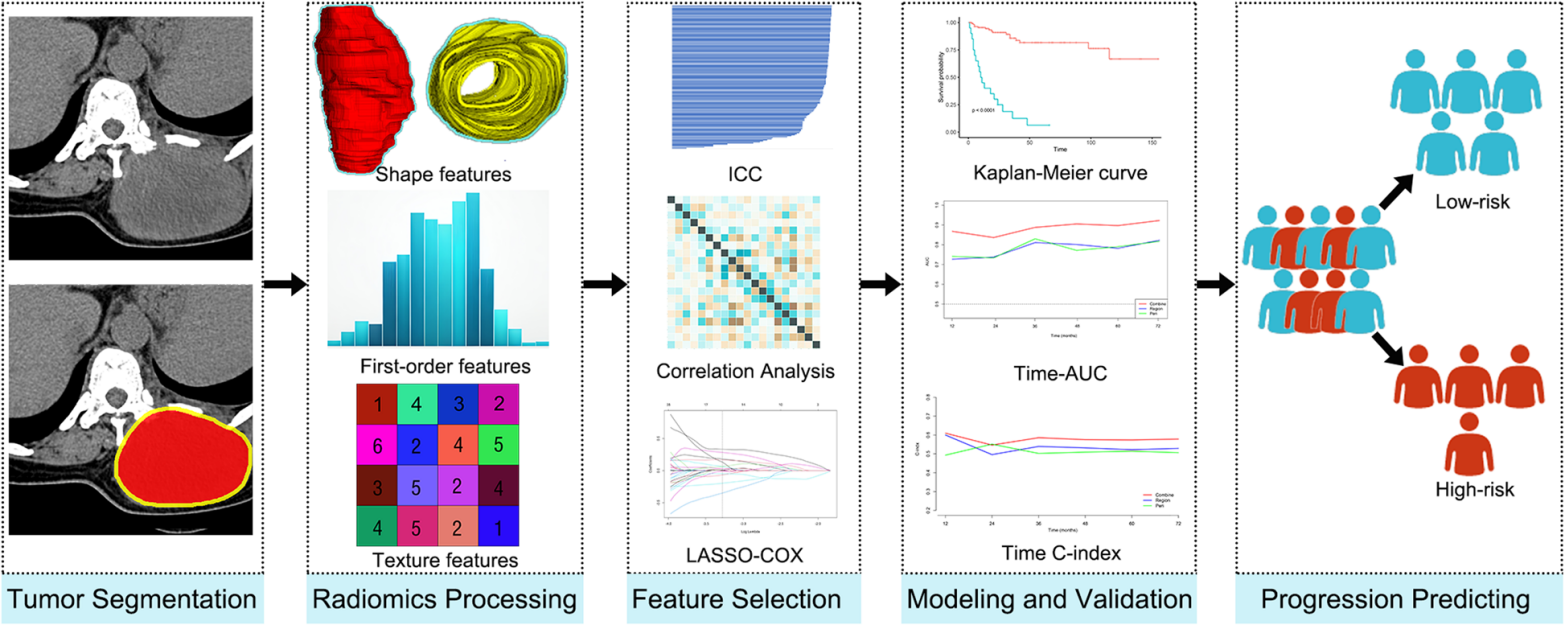
The workflow of the multicenter study. The tumor was segmented to determine the intratumoral ROI (red) and peritumoral ROI (yellow) from non-contrast-enhanced CT images. More related images of this case can be found in the supplementary material. ICC, intra-/inter-class correlation coefficient; LASSO, least absolute shrinkage and selection operator; AUC, area under the curve

The workflow of radiomics is shown in Fig. 2. The three-dimensional (3-D) region of interest of the tumor (ROI _region_) was segmented on axial CT images by two radiologists (Reader 1 and Reader 2, with 6 and 8 years of experience in interpretation of musculoskeletal imaging, respectively) using ITK-SNAP software (version 3.8.0, www.itksnap.org). The peritumoral region of interest (ROI _peri_) was generated by the “ROI operation” module of the RIAS software [24], which automatically expanded 3 mm outwards the tumor and removed the tumor area.

As the patients were from different centers, the CT images were resampled and grayscale discretized and normalized before feature extraction. The extraction of radiomics features was conducted through the RadCloud platform (Huiying Medical Technology Co., Ltd). A total of 2818 (1409 + 1409) radiomics features were extracted from the intratumoral and peritumoral ROIs, including first-order statistics, shape- and size-based features, texture features, and higher-order statistical features. The details of the radiomics features are shown in supplementary methods.

### Intra-observer and inter-observer reproducibility

Intra- and inter-class correlation coefficients (ICCs) were used to evaluate intra- and inter-observer reproducibility. Forty CT images were randomly selected, and ROI segmentation was performed independently by Reader 1 and Reader 2 to assess the inter-observer reproducibility. Reader 1 repeated the segmentation 2 weeks later to assess the intra-observer reproducibility. Intra- and inter-observer ICC > 0.75 indicated good reproducibility and radiomics features with ICC < 0.75 have been excluded [25]. The remaining image segmentations were performed by Reader 1.

### Development of intratumoral, peritumoral, and combined radiomics signatures

The selection of radiomics features was divided into three steps; both intratumoral and peritumoral radiomics features underwent the same process respectively. First, Pearson correlation analysis was used to reduce the redundancy of radiation signatures. Then, univariate Cox proportional regression analysis was used to select the radiomics features with *p <* 0.05. Finally, the least absolute shrinkage and selection operator (LASSO) Cox regression model was used to select the optimal features. *λ* was the regularization parameter of LASSO regression and was selected when the cross-validation error is minimum. Radiomics score (Rad-score) was calculated for each patient using a Cox proportional hazard regression model.

### Evaluation of intratumoral, peritumoral, and combined radiomics signatures

Harrell’s concordance index (C-index) and hazard ratio (HR) were used to assess the accuracy of the three radiomics signatures in predicting PFS in the training cohort and verified in the test cohort. X-tile software was used to determine the optimal threshold for Rad-score, and Kaplan-Meier survival analysis was used to analyze PFS in both groups to evaluate the prognostic significance of the three radiomics signatures. In order to compare the signature-predicted survival with the actual survival, calibration curves were generated. To investigate the prognostic performance of different radiomics signatures, time-dependent area under the receiver operating characteristics curve (time-AUC) and time-dependent C-index (time C-index) were used. The net reclassification improvement (NRI) was calculated to evaluate the incremental value of the combined signature over the single signature.

### Statistics

SPSS 26.0 software (IBM, Chicago, IL, USA) was used for independent sample Student *t*-test or Mann-Whitney *U*-test, chi-square (*χ*^2^) test, or Fisher exact test, where appropriate. ICC, Pearson correlation analysis, univariate Cox proportional regression analysis, LASSO Cox regression analysis, calibration plots, Kaplan-Meier survival analysis, C-index, time C-index, time-AUC analysis, and NRI analysis were performed with R statistical software (Version 4.2.1, https://www.r-project.org). A two-sided *p* < 0.05 was considered statistically significant.

## Results

### Clinical characteristics and follow-up

Table 1 shows the baseline characteristics of the 214 patients with CS. There was no significant difference in clinical and pathological characteristics between the training and test cohorts. During follow-up, 49 patients (22.9%) developed local recurrence or distant metastasis. The median PFS were 13 months (quartile range 5–34 months) and 40 months (quartile range 22.5–67.5 months) in patients with and without recurrence, respectively.

**Table 1:** Clinical characteristics of the patients with chondrosarcoma

Clinical characteristics	Training cohort (*n* = 113)	Test cohort (*n* = 101)	*P*
Age (mean ± SD), year	53.21 ± 13.811	51.25 ± 15.046	0.320
Sex (male/female)	58/55	48/56	0.322
Height (mean ± SD), cm	163.92 ± 15.841	160.10 ± 29.292	0.344
Weight (mean ± SD), kg	68.21 ± 12.416	66.09 ± 12.187	0.220
Location (extremity/other)	38/75	38/63	0.542
Tumor size (median [interquartile range]), mm	58 (42.97)	54 (32.79)	0.106
Surgery (yes/no)	100/13	95/6	0.153
Radiotherapy/chemotherapy (yes/no)	22/91	17/84	0.618
Histological grade (I/II & III)	58/54	63/39	0.144
PFS (median [interquartile range]), month	24 (8, 55)	26 (16, 45)	0.342

*SD*, standard deviation; *PFS*, progression-free survival

### Feature selection and construction of intratumoral, peritumoral, and combined radiomics signatures

Finally, 11, 7, and 16 radiomics features were selected to construct the intratumoral, peritumoral, and combined radiomics signatures (RS _region_, RS _peri_, RS _combine_), respectively. Details on feature selection and the formula for calculation of the Rad-score are shown in supplementary methods.

### Evaluation of intratumoral, peritumoral, and combined radiomics signatures

Table 2 shows estimates of C-index and HR associated with recurrence in different risk groups for each signature. RS _combine_ had the best predictive capacity, with a C-index of 0.800 (95% CI: 0.681–0.920) in the test cohort.

**Table 2:** The performance of the RS _region_, RS _peri_, and RS _combine_ in predicting PFS of the patients with chondrosarcoma

Model	Training cohort		Test cohort	
	C-index (95% CI)	HR (95% CI)	C-index (95% CI)	HR (95% CI)
RS _region_	0.733 (0.645–0.820)	5.574 (2.653–11.712)	0.722 (0.585–0.860)	13.095 (2.788–61.518)
RS _peri_	0.716 (0.621–0.811)	28.712 (8.676–95.011)	0.705 (0.551–0.858)	19.824 (3.870–101.550)
RS _combine_	0.835 (0.764–0.905)	228.754 (70.068–746.830)	0.800 (0.681–0.920)	11.841 (3.337–42.025)

*RS*, radiomics signature; C-index, concordance index; *HR*, hazard ratio; *CI*, confidence interval

**Fig. 3:**
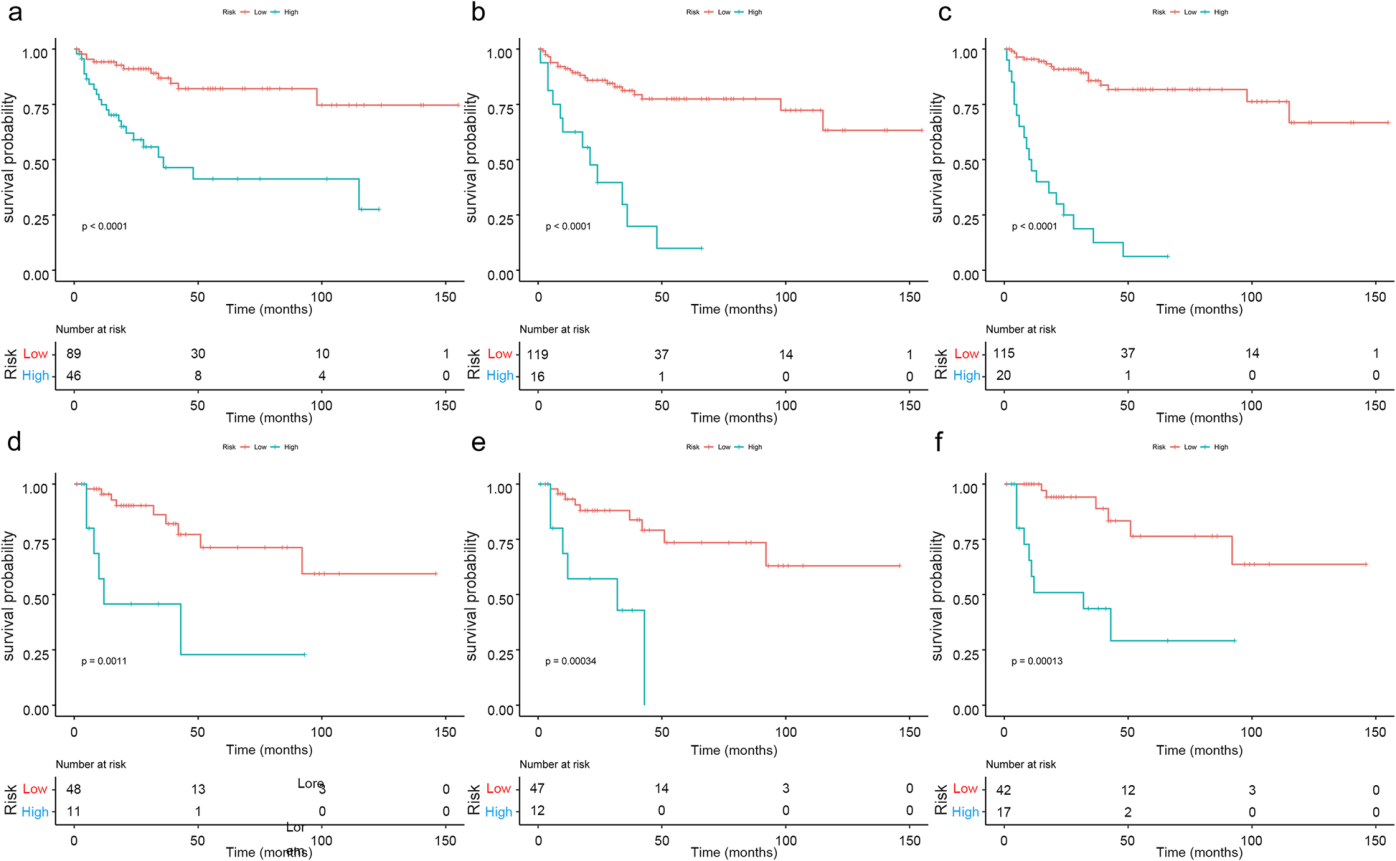
Kaplan-Meier survival curves for progression-free survival (PFS) by the intratumoral radiomics signature (RS _region_; **a** training cohort; **d** test cohort), peritumoral radiomics signature (RS _peri_; **c** training cohort; **e** test cohort), and combined radiomics signature (RS _combine_; **c** training cohort; **f** test cohort), respectively, in patients with chondrosarcoma

**Fig. 4:**
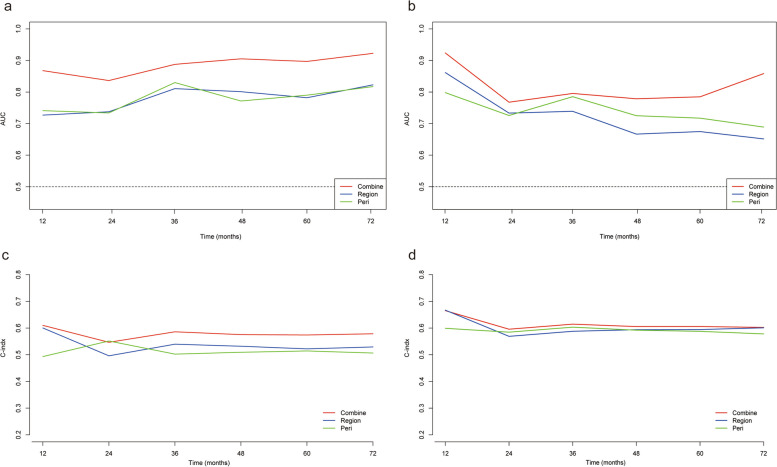
The time-area under the curve (time-AUC; **a** training cohort; **b** test cohort) and time-dependent C-index (**c** training cohort; **d** test cohort) of the intratumoral radiomics signature (RS _region_), the peritumoral radiomics signature (RS _peri_), and the combined radiomics signature (RS _combine_) in the prediction of progression-free survival (PFS) in patients with chondrosarcoma

Kaplan-Meier survival analysis showed that the Rad-score calculated by the three signatures was correlated with PFS (Fig. 3). The calibration curves of each signature are shown in Fig. S3. Compared with RS _region_ and RS _peri_, RS _combine_ showed higher time-AUCs and time-dependent C-indices (Fig. 4). Compared with the RS _region_, the combined signature provided an NRI of 0.297 (95% CI: 0.119–0.442, *p* < 0.001). The RS _combine_ provided an NRI of 0.217 (95% CI: 0.103–0.418, *p* = 0.03) when compared with the peritumoral signature.

## Discussion

In this retrospective multicenter study, we developed and validated CT-based intratumoral, peritumoral, and combined radiomics signatures to predict PFS in patients with CS. We evaluated the predictive performance of the three signatures and found that all three signatures have the ability to classify high-risk patients from low-risk ones for progression; the combined radiomics signature provided higher predictive values than the other two single radiomics signatures for the outcome of CS patients.

CS is the second most common primary malignant bone tumor. More than 90% of CS are low-to-intermediate-grade tumors, and 5–10% of CS are high-grade aggressive tumors with a high propensity to metastasize [26, 27]. Accurate prediction of outcomes for CS patients is of great importance as it may facilitate clinicians in selecting proper surveillance and treatment strategies, thus improving the prognosis of CS patients. Several prognostic factors associated with recurrence have been identified, such as tumor size, grade, and stage [1, 28, 29]. The preoperative evaluation of bone tumors usually requires a combination of clinical features, imaging findings, and histopathological findings. With the rapid development of artificial intelligence, the power and potential of big image data are increasingly recognized in the field of oncology [13]. Yin et al. developed a nomogram based on 3D MR radiomics and clinical features for the assessment of early recurrence of pelvic CS; they found that the combined nomogram was superior to the clinical model (AUC: 0.891 vs. 0.625), and the Rad-score was the most important independent risk factor in predicting early recurrence of pelvic CS (OR = 3, *p* < 0.01) [21]. Our study also verified the association between radiomics-based tumor heterogeneity and PFS in patients with CS. The C-index of the RS _region_ in the test cohort was 0.722, indicating a favorable predictive value of the intratumoral radiomics signature for CS patients.

The changes in the stroma surrounding the tumor determine the ability of the tumor to grow and spread, evade the body’s immune protection, and resist therapeutic interventions [30]. In addition, evidence suggests that peritumoral areas are reflecting peritumoral immune cell infiltration [31, 32]. Therefore, peritumoral heterogeneity and microenvironment are also associated with the aggressive behaviors of tumors. However, the features of the peritumoral areas cannot be effectively characterized by radiomics analysis of the tumor parenchyma. Nowadays, many studies have integrated the peritumoral radiomics features into intratumoral radiomics models or clinical models in survival analysis, such as esophageal carcinoma [22], breast cancer [33], hepatocellular carcinoma [34], and lung cancer [35]. Hu et al. demonstrated that a combined model consisting of intra- and peri-tumoral radiomics features could predict pathological complete response in patients with esophageal squamous cell carcinoma after neoadjuvant chemoradiotherapy, with an AUC of 0.852 (95% CI, 0.753–0.951) and the accuracy of 84.3% in the test cohort [22]. Khorrami et al. revealed that the intra- and peritumoral shape and texture features extracted from CT images could identify pathological response to neoadjuvant chemoradiotherapy in patients with non-small cell lung cancer with AUCs of 0.90 and 0.86 in the training cohort and the test cohort. In addition, the radiomics features were also significantly associated with OS (HR, 11.18%; 95% CI, 3.17–44.1) and PFS (HR, 2.78; 95% CI, 1.11–4.12) [36]. Chong et al. developed a peritumoral radiomic model using liver-biliary-specific Gd-EOB-DTPA MRI in patients with hepatocellular carcinoma (HCC) and found this model provided the best clinical net benefit (NRI: 35.9–66.1%, *p* < 0.001) and was substantially more efficient than the existing clinical algorithms in predicting early recurrence of HCC [37].

Sufficient characterization of intra- and peritumoral heterogeneity and microenvironment contributes to precise outcome prediction for CS. Although intra- and peritumoral radiomics features represent different spatially heterogeneous information, they are not redundant but complementary. In this study, the CT-based peritumoral radiomics signature yielded a C-index of 0.705 in predicting the PFS of the patients with CS in the test cohort. By incorporating the peritumoral radiomics features into the intratumoral ones, the RS _combine_ achieved a higher C-index (0.800, in the test cohort), time-AUC, and NRI than the RS _region_ and RS _peri_ alone, indicating the incremental value of the peritumoral heterogeneity and microenvironment to the intratumoral radiomics in individualized survival estimation in CS. Therefore, we believe that intratumoral and peritumoral radiomics features can be incorporated, which may provide additional predictive value for survival in patients with CS. Guided by the combined signature, if the patients are stratified as high risk for progression, intensive surveillance and systemic adjuvant therapy will be recommended. On the contrary, only regular surveillance is advocated for the RS _combine_-predicted low-risk patients.

Admittedly, there were some limitations to this study. First, since the patients come from different centers, there was an inevitable difference in scanner parameters. To avoid the impact of this discrepancy, the radiomics analysis should be standardized before its implementation in clinical practice, including image acquisition, feature extraction, and data processing. Second, in the current study, 3D segmentations of the tumors, which were manually performed, were challenging and time-consuming. The development of advanced machine learning methods for semiautomated or fully automated tumor segmentation is likely to drive its wide application in the future. Third, only about 30% of CS patients were enrolled in our study according to the inclusion and exclusion criteria. This is because our study was a retrospective study, which could not carry out the homogeneous management of patients. Patients should be better managed to address this issue in future applications. We will use larger patient cohorts and establish prognostic models based on CT, MR, and radiograph respectively in the future study to apply to different patient populations.

In conclusion, our study revealed that the intratumoral and peritumoral radiomics features are potential prognostic biomarkers in CS patients. The combined radiomics signature incorporating the intratumoral and peritumoral radiomics features may serve as a novel imaging tool to predict the prognosis of patients with CS, thus improving individualized treatment and management of CS patients and further promoting precision medicine.

### Supplementary Information

**Additional file 1:** **Appendix S1.** The radiomics features can be divided into four groups: (1) intensity statistic features, which consists of 19 features that quantitatively delineate the distribution of voxel intensities within the ROIs through commonly used and basic metrics; (2) shape features, including 14 3-D features, are used to reflect the shape and size of the ROIs; (3) texture features, are composed of 59 features calculated by gray level co-occurrence matrix (GLCM), gray level run length matrix (GLRLM) and gray level size zone matrix (GLSZM), quantifying the heterogeneity differences of ROIs; and (4) filter and wavelet features, which include the intensity and texture features derived from filter transformation and wavelet transformation of the original images, obtained by applying filters such exponential, logarithm, square, square root and wavelet (wavelet-LHL, wavelet-LHH, wavelet-HLL, wavelet-LLH, wavelet-HLH, wavelet-HHH, wavelet-HHL and wavelet-LLL). **Appendix S2.** Among the 2818 radiomics features extracted from the CT images, 1778 repeatable and stable radiomics with ICCs > 0.75 were retained, including 1154 intratumoral and 624 peritumoral radiomics features. **Fig. S1.** Chondrosarcoma of the right transverse process of thoracic vertebra in a 58-year-old woman. **Fig. S2.** Feature selection for the development of the intratumoral radiomics signature (RS _region_, a), peritumoral radiomics signature (RS _peri_, b), and combined radiomics signature (RS _combine_, c), respectively using the least absolute shrinkage and selection operator regression model with a vertical line generated at the log (λ) value by using tenfold cross-validation. The 11 intratumoral radiomics features (d), 7 peritumoral radiomics features (e), and 16 intra-/peritumoral radiomics features (f) and their corresponding coefficients. **Fig. S3.** The calibration curves of the intratumoral radiomics signature (a, training cohort; b, test cohort), the peritumoral radiomics signature (c, training cohort; d, test cohort), the combined radiomics signature (e, training cohort; f, test cohort), for predicting 1-, 3- and 5- year progression-free survival (PFS) in patients with chondrosarcoma, respectively. **Table S1.** CT scan protocols.

## Data Availability

The datasets used and/or analyzed during the current study are available from the corresponding author on reasonable request.

## Abbreviations

3-D: Three-dimensional
AUC: Area under the curve
CS: Chondrosarcoma
ER: Early recurrence
HR: Hazard ratio
ICC: Intra-/inter-class correlation coefficient
LASSO: Least absolute shrinkage and selection operator
mpMRI: Multiparameter magnetic resonance imaging
NRI: Net reclassification improvement
OR: Odds ratio
OS: Overall survival
PFS: Progression-free survival
Rad-score: Radiomics score
ROI: Region of interest
SEER: Surveillance, Epidemiology, and End Results
