# Supplementary material for: Intratumoral and peritumoral CT radiomics in predicting prognosis in patients with chondrosarcoma: a multicenter study
Source: Insights Imaging. 2024 Jan 17;15:9. doi: 10.1186/s13244-023-01582-8 (PMC10792153; doi:10.1186/s13244-023-01582-8)
Supplement: Supplementary file 1 — Additional file 1: Appendix S1. The radiomics features can be divided into four groups: (1) intensity statistic features, which consists of 19 features that quantitatively delineate the distribution of voxel intensities within the ROIs through commonly used and basic metrics; (2) shape features, including 14 3-D features, are used to reflect the shape and size of the ROIs; (3) texture features, are composed of 59 features calculated by gray level co-occurrence matrix (GLCM), gray level run length matrix (GLRLM) and gray level size zone matrix (GLSZM), quantifying the heterogeneity differences of ROIs; and (4) filter and wavelet features, which include the intensity and texture features derived from filter transformation and wavelet transformation of the original images, obtained by applying filters such exponential, logarithm, square, square root and wavelet (wavelet-LHL, wavelet-LHH, wavelet-HLL, wavelet-LLH, wavelet-HLH, wavelet-HHH, wavelet-HHL and wavelet-LLL). Appendix S2. Among the 2818 radiomics features extracted from the CT images, 1778 repeatable and stable radiomics with ICCs > 0.75 were retained, including 1154 intratumoral and 624 peritumoral radiomics features. Fig. S1. Chondrosarcoma of the right transverse process of thoracic vertebra in a 58-year-old woman. Fig. S2. Feature selection for the development of the intratumoral radiomics signature (RS region, a), peritumoral radiomics signature (RS peri, b), and combined radiomics signature (RS combine, c), respectively using the least absolute shrinkage and selection operator regression model with a vertical line generated at the log (λ) value by using tenfold cross-validation. The 11 intratumoral radiomics features (d), 7 peritumoral radiomics features (e), and 16 intra-/peritumoral radiomics features (f) and their corresponding coefficients. Fig. S3. The calibration curves of the intratumoral radiomics signature (a, training cohort; b, test cohort), the peritumoral radiomics signature (c, training coh [file 13244_2023_1582_MOESM1_ESM.docx]

**Intratumoral and peritumoral CT radiomics in predicting prognosis in patients with chondrosarcoma: a multicenter study**

**ELECTRONIC SUPPLEMENTARY MATERIAL**

**Appendix S1**

The radiomics features can be divided into four groups: (1) intensity statistic features, which consists of 19 features that quantitatively delineate the distribution of voxel intensities within the ROIs through commonly used and basic metrics; (2) shape features, including 14 3-D features, are used to reflect the shape and size of the ROIs; (3) texture features, are composed of 59 features calculated by gray level co-occurrence matrix (GLCM), gray level run length matrix (GLRLM) and gray level size zone matrix (GLSZM), quantifying the heterogeneity differences of ROIs; and (4) filter and wavelet features, which include the intensity and texture features derived from filter transformation and wavelet transformation of the original images, obtained by applying filters such exponential, logarithm, square, square root and wavelet (wavelet-LHL, wavelet-LHH, wavelet-HLL, wavelet-LLH, wavelet-HLH, wavelet-HHH, wavelet-HHL and wavelet-LLL).

**Appendix S2**

Among the 2818 radiomics features extracted from the CT images, 1778 repeatable and stable radiomics with ICCs > 0.75 were retained, including 1154 intratumoral and 624 peritumoral radiomics features.

Construction of intratumoral radiomics signature: Firstly, 253 radiomics features were selected by Pearson correlation analysis. Then 135 radiomics features associated with RFS by a univariate Cox analysis were entered into the LASSO Cox regression model. Finally, 11 most valuable features were selected to develop the radiomics signature. The formula of the Rad-score is as follows.

Rad-score= -0.080×wavelet.HLL_glcm_region_Autocorrelation+ 0.258×wavelet.LHH_glszm_region_GrayLevelNonUniformity+

0.042×wavelet.HHH_glszm_region_SizeZoneNonUniformity+

0.224×wavelet.HLH_firstorder_region_Maximum+

0.072×wavelet.HLL_firstorder_region_Kurtosis-0.005×wavelet.LLH_glcm_region_Imc2-0.134×wavelet.LLL_glcm_region_ClusterShade-0.003×wavelet.LLL_glszm_region_ZonePercentage-0.249×wavelet.HHL_glcm_region_JointEnergy-0.046×wavelet.LHH_glcm_region_Autocorrelation+

0.195043×wavelet.HHH_firstorder_region_TotalEnergy

Construction of peritumoral radiomics signature: Firstly, 132 radiomics features were selected by Pearson correlation analysis. Then 55 radiomics features associated with RFS by a univariate Cox analysis were entered into the LASSO Cox regression model. Finally, 7 most valuable features were selected to develop the radiomics signature. The formula of the Rad-score is as follows.

Rad-score=0.0112×wavelet.LLH_glszm_peri_ZoneEntropy+

0.234×wavelet.LHH_firstorder_peri_Energy+

0.094×wavelet.HLL_firstorder_peri_Kurtosis+

0.059×wavelet.LHH_firstorder_peri_TotalEnergy+

0.109×wavelet.HLH_glszm_peri_SizeZoneNonUniformity-0.124×wavelet.HLL_firstorder_peri_Minimum+

0.063×square_firstorder_peri_Kurtosis

Construction of combined radiomics signature: Firstly, 308 radiomics features were selected by Pearson correlation analysis. Then 293 radiomics features associated with RFS by a univariate Cox analysis were entered into the LASSO Cox regression model. Finally, 16 most valuable features were selected to develop the radiomics signature, including 8 intratumoral radiomics features and 8 peritumoral radiomics features. The formula of the Rad-score is as follows.

Rad-score=0.180×wavelet.HHH_glszm_region_SizeZoneNonUniformity+

0.006×wavelet.HLH_firstorder_region_Maximum+

0.031×wavelet.HLL_firstorder_region_Kurtosis-0.196×wavelet.LLL_glcm_region_ClusterShade-0.009×gradient_firstorder_region_90Percentile-0.347×wavelet.HHL_glcm_region_JointEnergy+

0.129×wavelet.LHH_glcm_region_ClusterShade+

0.063×wavelet.HHH_firstorder_region_TotalEnergy+

0.031×wavelet.HLH_firstorder_peri_Skewness+

0.332×wavelet.LHH_firstorder_peri_Energy+

0.166×wavelet.HLL_firstorder_peri_Kurtosis+

0.066×wavelet.LHH_firstorder_peri_TotalEnergy-0.066×wavelet.LHH_firstorder_peri_Minimum-0.049×wavelet.HLL_firstorder_peri_Minimum+

0.283×square_firstorder_peri_Kurtosis+

0.008×wavelet.HLH_firstorder_peri_Maximum

**Table S1 CT scan protocols**

| **CT scanner** | **CT 64** | **CT 64** | **CT 64** | **CT 64** | **CT 16** | **CT 16** |
| --- | --- | --- | --- | --- | --- | --- |
| Scanner model | Somatom Sensation 64 | Discovery 750 | Somatom Definition | LightSpeed VCT | Brilliance 16 | Brightspeed 16 |
| Manufacturer | Siemens | General Electric | Siemens | General Electric | Philips | General Electric |
| Gantry rotation time (s) | 0.5 | 0.5 | 0.5 | 0.5 | 0.5 | 0.5 |
| Tube voltage (kV) | 120 | 120 | 120- | 120 | 120 | 120 |
| Tube current | 200 mAs  (automatic tube current modulation) | 150-450 mA (automatic tube current modulation) | 200 mAs  (automatic tube current modulation) | 200 mA  (automatic tube current modulation) | 200 mAs (automatic tube current modulation) | 200 mA (automatic tube current modulation) |
| Detector collimation (mm) | 0.6 | 0.625 | 0.6 | 0.625 | 0.75 | 0.625 |
| Matrix | 512×512 | 512×512 | 512×512 | 512×512 | 512×512 | 512×512 |
| Pitch | 1.0 | 1.375 | 1.25 | 0.984 | 1 | 1.25 |
| Slice thickness (mm) | 5 | 5 | 5 | 5 | 5 | 5 |
| reconstruction kernel | B30f | Standard | B30f | Standard | B | Standard |
| Hospital | a, b | a, b | a, b | a, b | a | b |

NOTE: s (second), kV (kilovolt), mA (milliampere), mm (milimetre). a. The Affiliated Hospital of Qingdao University; b. Shandong Provincial Hospital Affiliated to Shandong First Medical University


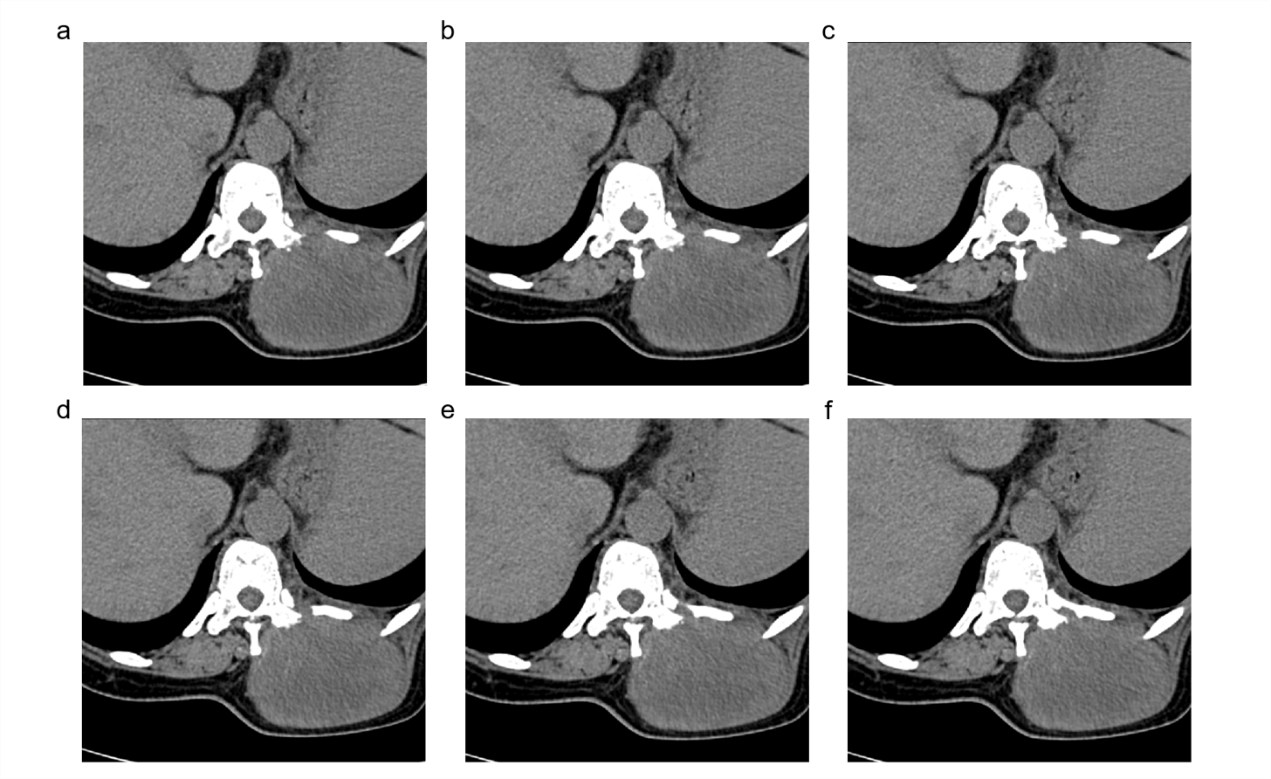


**Fig. S1** Chondrosarcoma of the right transverse process of thoracic vertebra in a 58-year-old woman.


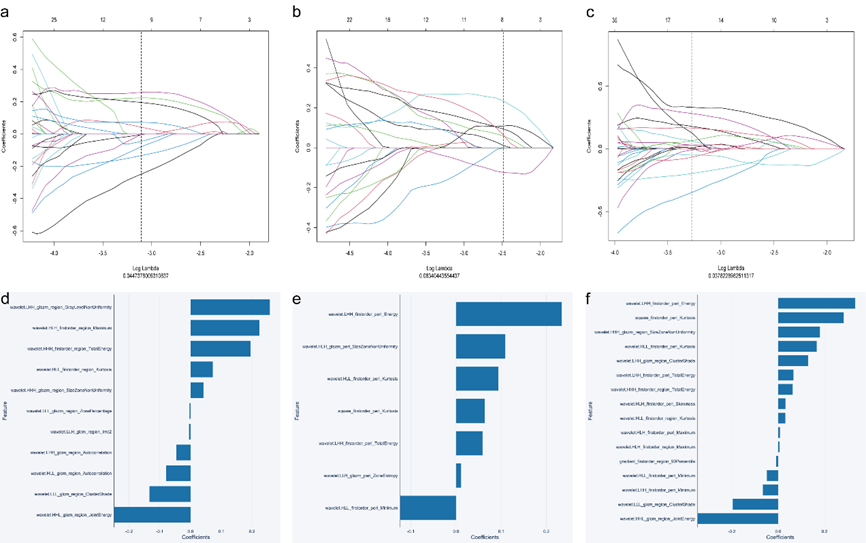


**Fig. S2** Feature selection for the development of the intratumoral radiomics signature (RS _region_, a), peritumoral radiomics signature (RS _peri_, b), and combined radiomics

signature (RS _combine_, c), respectively using the least absolute shrinkage and selection operator regression model with a vertical line generated at the log (λ) value by using tenfold cross-validation. The 11 intratumoral radiomics features (d), 7 peritumoral radiomics features (e), and 16 intra-/peritumoral radiomics features (f) and their corresponding coefficients.


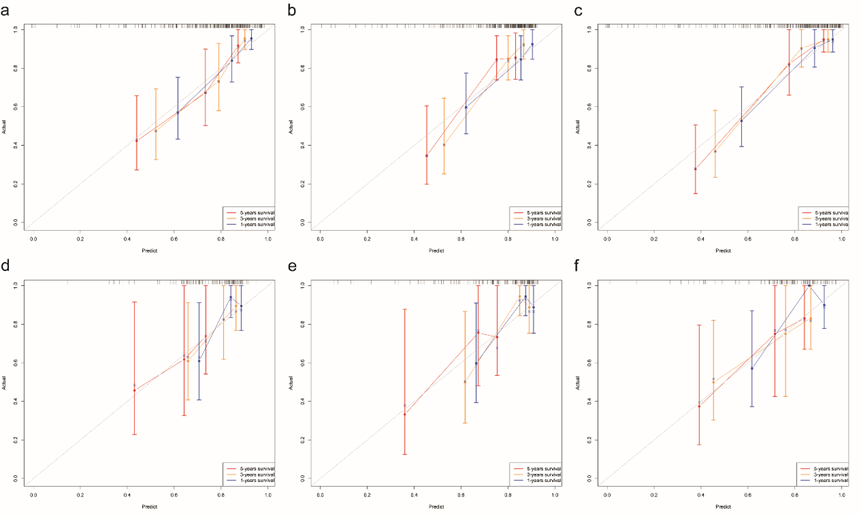


**Fig. S3** The calibration curves of the intratumoral radiomics signature (a, training cohort; b, test cohort), the peritumoral radiomics signature (c, training cohort; d, test cohort), the combined radiomics signature (e, training cohort; f, test cohort), for predicting 1-, 3- and 5- year progression-free survival (PFS) in patients with chondrosarcoma, respectively.
